# Kidney health for all: bridging the gap in kidney health education and literacy

**DOI:** 10.1007/s40620-022-01290-4

**Published:** 2022-03-14

**Authors:** Robyn G. Langham, Kamyar Kalantar-Zadeh, Ann Bonner, Alessandro Balducci, Li-Li Hsiao, Latha A. Kumaraswami, Paul Laffin, Vassilios Liakopoulos, Gamal Saadi, Ekamol Tantisattamo, Ifeoma Ulasi, Siu-Fai Lui, For the World Kidney Day Joint Steering Committee, Robyn G Langham, Kamyar Kalantar-Zadeh, Alessandro Balducci, Li-Li Hsiao, Latha Kumaraswami, Paul Laffin, Vassilios Liakopoulos, Gamal Saadi, Ifeoma Ulasi, Siu-Fai Lui

**Affiliations:** 1 Department of Medicine, St. Vincent’s Hospital, University of Melbourne, Melbourne, VIC Australia; 2 Division of Nephrology, Hypertension and Kidney Transplantation, Department of Medicine, University of California Irvine School of Medicine, Orange, CA USA; 3 School of Nursing and Midwifery, Griffith University, Southport, QLD Australia; 4 Italian Kidney Foundation, Rome, Italy; 5 Renal Division, Department of Medicine, Brigham and Women’s Hospital, Boston, MA USA; 6 Tamilnad Kidney Research (TANKER) Foundation, The International Federation of Kidney Foundations-World Kidney Alliance (IFKF-WKA), Chennai, India; 7 International Society of Nephrology, Brussels, Belgium; 8 Division of Nephrology and Hypertension, 1st Department of Internal Medicine, AHEPA Hospital, Aristotle University of Thessaloniki, Thessaloniki, Greece; 9 Nephrology Unit, Department of Internal Medicine, Faculty of Medicine, Cairo University, Giza, Egypt; 10 Renal Unit, Department of Medicine, College of Medicine, University of Nigeria, Ituku-Ozalla, Enugu, Nigeria; 11 The Jockey Club School of Public Health and Primary Care, International Federation of Kidney Foundations—World Kidney Alliance, The Chinese University of Hong Kong, Hong Kong, China

**Keywords:** Educational gap, Empowerment, Health literacy, Health policy, Information technology, Kidney health, Partnership, Prevention, Social media

## Abstract

The high burden of kidney disease, global disparities in kidney care, and poor outcomes of kidney failure bring a concomitant growing burden to persons affected, their families, and carers, and the community at large. Health literacy is the degree to which persons and organizations have or equitably enable individuals to have the ability to find, understand, and use information and services to make informed health-related decisions and actions for themselves and others. Rather than viewing health literacy as a patient deficit, improving health literacy largely rests with health care providers communicating and educating effectively in codesigned partnership with those with kidney disease. For kidney policy makers, health literacy provides the imperative to shift organizations to a culture that places the person at the center of health care. The growing capability of and access to technology provides new opportunities to enhance education and awareness of kidney disease for all stakeholders. Advances in telecommunication, including social media platforms, can be leveraged to enhance persons’ and providers’ education; The World Kidney Day declares 2022 as the year of “*Kidney Health for All*” to promote global teamwork in advancing strategies in bridging the gap in kidney health education and literacy. Kidney organizations should work toward shifting the patient-deficit health literacy narrative to that of being the responsibility of health care providers and health policy makers. By engaging in and supporting kidney health–centered policy making, community health planning, and health literacy approaches for all, the kidney communities strive to prevent kidney diseases and enable living well with kidney disease.

**Supplementary Information:**

The online version contains supplementary material available at 10.1007/s40620-022-01290-4.

Given the high burden of kidney disease and global disparities related to kidney care, in carrying forward our mission of advocating *Kidney Health for All*, the challenging issue of bridging the well-identified gap in the global understanding of kidney disease and its health literacy is the theme for World Kidney Day (WKD) 2022. Health literacy is defined as the degree to which persons and organizations have—or equitably enable individuals to have—the ability to find, understand, and use information and services to inform health-related decisions and actions for themselves and others [1]. Not only is there growing recognition of the role that health literacy has in determining outcomes for persons affected by kidney disease and the community in general, but there is an emergent imperative for policy makers worldwide to be informed and cognizant of opportunities and real measurable outcomes that can be achieved through kidney-specific preventative strategies.

## The global community of people with kidney disease

Most people are not aware of what kidneys are for or even where their kidneys are. For those afflicted by disease and the subsequent effects on overall health, an effective health care provider communication is required to support individuals to be able to understand what to do, to make decisions, and to take action. Health literacy involves more than functional abilities of an individual; it is also the cognitive and social skills needed to gain access to, understand, and use information to manage health condition [2]. It is also contextual [3] in that as health needs change, so too does the level of understanding and ability to problem solve alter. Health literacy is, therefore, an interaction between individuals, health care providers, and health policy makers [4]. This why the imperatives around health literacy are now recognized as indicators for the quality of local and national health care systems and health care professionals within it [5]. For chronic kidney disease (CKD), as the disease progresses alongside other health changes and increasing treatment complexities, it becomes more difficult for individuals to manage [6]. Promoted in health policy for around a decade involving care partnerships between health-centered policy, community health planning, and health literacy [7], current approaches need to be shifted forward (Table 1).

**Table 1:** Summary characteristic of kidney health promotion, involving kidney health–centered policy, community kidney health planning, and kidney health literacy, and proposed future direction

Kidney health promotion	Definition	Stakeholders	Current status	Limitations/challenges	Suggested solutions/future research
Kidney health–centered policy	Incorporate kidney health into policy decision making Prioritize policies with primary prevention for CKD	Governance Policy makers Insurance agencies	Policy emphasizing treatment for CKD and kidney failure rather than kidney health prevention	Economic-driven situation challenging CKD risk factor minimization (e.g., food policy)	Promote implementation of public health program for primary CKD prevention Promote sustainable treatment for CKD and dialysis Increase kidney transplant awareness Enhance visibility and encourage brother-sister nephrology and transplant program in LMIC Support research funding from government Health care cost-effectiveness for caring for CKD Kidney failure, including maintenance dialysis and transplant Promote surveillance programs for kidney diseases and their risk factors
Community kidney health planning	Building up preventive strategies to promote healthy communities and primary health care facilities	Community leadership Kidney patient advocacy	Belief in community leaders in LMIC	Education and understanding kidney health promotion of community leadership and people	Improve role model of community Enhance kidney support networks
Kidney health literacy	Receive knowledge, skills and information to be healthy	People with CKD Care partners Health care providers	Lack of awareness of CKD and risk factors Care partner burden and burnout Inadequate health care workers High patients-to-health care workers ratio, especially in rural areas	Inadequate policy direction Ineffective health care providers’ communication skills	Organizational paradigm shift toward health literacy Improving communication between health care providers with patients and care partners Using teach-back methods for consumer education Adapting technologies for appropriate health literacy and sociocultural environments Family engagement in the patient care Incentive for community health care providers in rural areas

*CKD* chronic kidney disease, *LMIC* low- to middle-income country

Assessing health literacy necessitates the use of appropriate multidimensional patient-reported measures, such as the World Health Organization–recommended Health Literacy Questionnaire (available in over 30 languages) rather than tools measuring only functional health literacy (e.g., Rapid Estimate of Adult Literacy in Medicine or Short Test of Functional Health Literacy in Adults) [8]. It is therefore not surprising that studies of low health literacy (LHL) abilities in people with CKD have been demonstrated to be associated with poor CKD knowledge, self-management behaviors, and health-related quality of life and in those with greater comorbidity severity [7]. Unfortunately, most CKD studies have measured only functional health literacy, so the evidence that LHL results in poorer outcomes, particularly that it increases health care utilization and mortality [9], and reduces access to transplantation [10], is weak.

Recently, health literacy is now considered to be an important bridge between lower socioeconomic status and other social determinants of health [4]. Indeed, this is not a feature that can be measured by the gross domestic product of a country, as the effects of LHL on the extent of CKD in the community are experienced globally regardless of country income status. The lack of awareness of risk factors of kidney disease, even in those with high health literacy abilities, is testament to the difficulties in understanding this disease, and why the United States, for instance, recommends that a universal precautions approach toward health literacy is undertaken. [11].

So, what does the perfect health literacy program look like for people with CKD? In several high-income countries, there are national health literacy action plans with the emphasis shifted to policy directives, organizational culture, and health care providers. In Australia, for instance, a compulsory health literacy accreditation standard makes the health care organization responsible for ensuring providers are cognizant of individual health literacy abilities [12]. Although many high-income countries, health care organizations, nongovernmental organizations, and jurisdictions are providing an array of consumer-facing web-based programs that provide detailed information and self-care training opportunities, most are largely designed for individual/family use that are unlikely to mitigate LHL. There is, however, substantial evidence that interventions improving health care provider communication are more likely to improve understanding of health problems and abilities to adhere to complex treatment regimens. [13].

Access to information that is authentic and tailored specifically to the needs of the individual and the community is the aim. The challenge is recognized acutely in more remote and low- to middle-income countries of the world, specifically the importance of culturally appropriate knowledge provision. The principles of improving health literacy are the same, but understanding how to proceed, and putting consumers in charge, with a codesign approach, is critical and may result in a different outcome in more remote parts of the world. This principle especially applies to communities that are smaller, with less access to electronic communication and health care services, where the level of health literacy is shared across the community and where what affects the individual also affects all the community. Decision support systems are different, led by elders, and in turn educational resources are best aimed at improving knowledge of the whole community.

A systematic review of the evaluation of interventions and strategies shows this area of research is still at an early stage [14], with no studies unraveling the link between LHL and poor CKD outcomes. The best evidence is in supporting targeted programs on improving communication capabilities of health care professionals as central. One prime example is Teach-back, a cyclical, simple, low-cost education intervention that shows promise for improving communication, knowledge, and self-management in the CKD populations in low- or high-income countries [15]. Furthermore, the *consumer*-led voice has articulated research priorities that align closely with principles felt to be important to the success of education: building new education resources, devised in partnership with consumers, and focused on the needs of vulnerable groups. Indeed, programs that address the lack of culturally safe, person-centered and holistic care, along with improving the communication skills of health professionals, are crucial for those with CKD. [16].

## The networked community of kidney health care workers

**Fig. 1:**
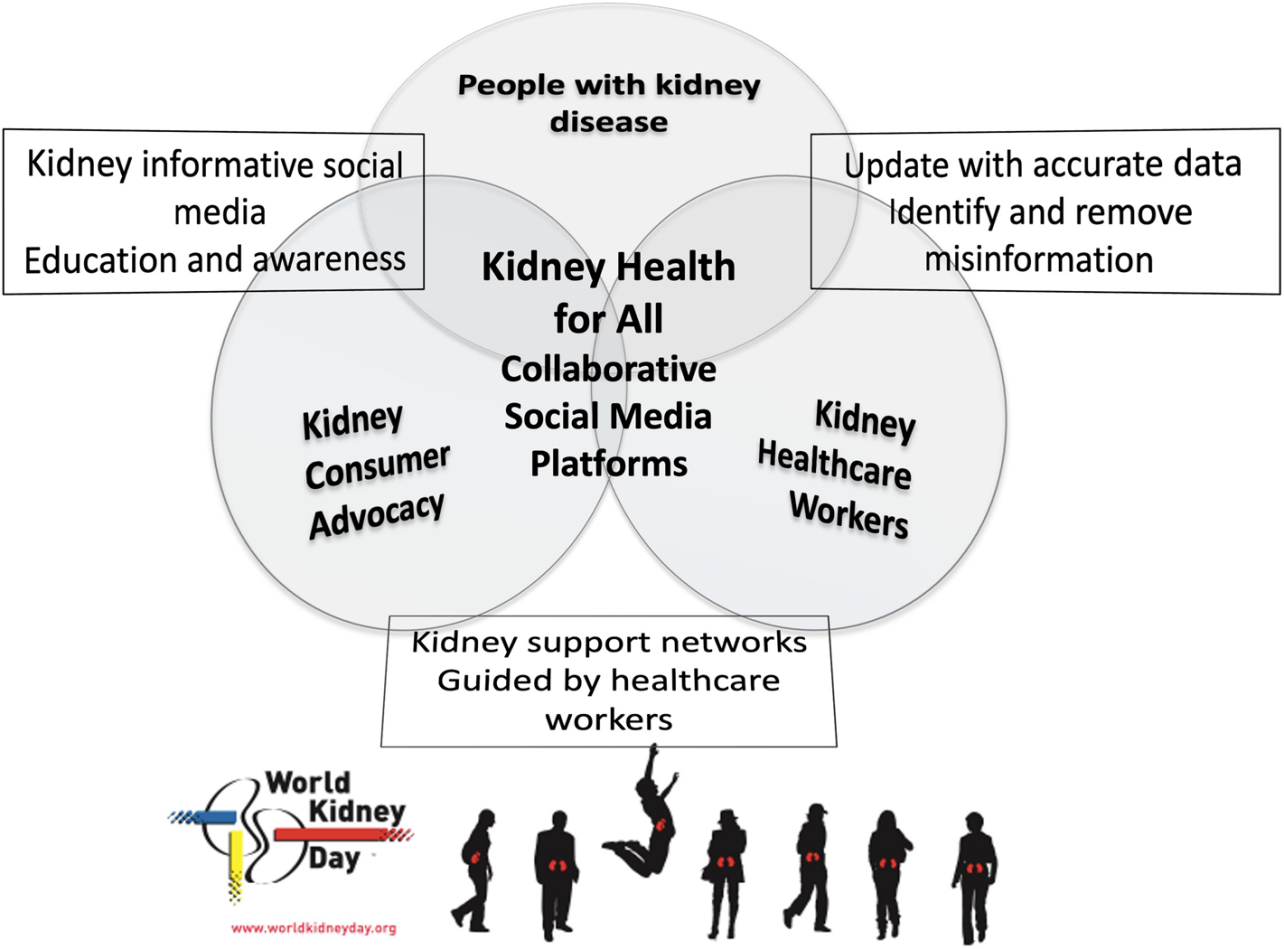
Schematic representation of consumer and health care professionals’ collaborative advocacy using social media platforms with the goal of *Kidney Health for All*

Nonphysician health care workers, including nurses and advanced practice providers (physician assistants and nurse practitioners) as well as dietitians, pharmacists, social workers, technicians, physical therapists, and other allied health professionals, often spend more time with persons with kidney disease, compared with nephrologists and other physician specialists. In an ambulatory care setting at an appointment, in the emergency department, or in the inpatient setting, these health care professionals often see and relate to the patient first, last, and in between, given that physician encounters are often short and focused. Hence, the nonphysician health care workers have many opportunities to discuss kidney disease–related topics with the individuals and their care partners and to empower them [17, 18]. For instance, medical assistants can help identify those with or at risk of developing CKD and can initiate educating them and their family members about the role of diet and lifestyle modification for primary, secondary, and tertiary prevention of CKD while waiting to see the physician [19]. Some health care workers provide networking and support for kidney patient advocacy groups and kidney support networks, which have been initiated or expanded via social media platforms (Fig. 1) [20, 21]. Studies examining the efficacy of social media in kidney care and advocacy are on the way. [22, 23].

Like physicians, many activities of nonphysician health care workers have been increasingly affected by the rise of electronic health recording and growing access to internet-based resources, including social media, that offer educational materials related to kidney health, including kidney-preserving therapies with traditional and emerging interventions [24]. These resources can be used for both self-education and for networking and advocacy on kidney disease awareness and learning. Increasingly, more health care professionals are engaged in some types of social media–based activities, as shown in Table 2. At the time of this writing, the leading social media used by many—but not all—kidney health care workers include Facebook, Instagram, Twitter, LinkedIn, and YouTube. In some regions of the world, certain social media are more frequently used than others given unique cultural or access constellations (e.g., WeChat is a platform often used by health care workers and patient groups in China). Some health care professionals, such as managers and those in leadership and advocacy organization positions, may choose to embark on social media to engage those with CKD and their care partners or other health care professionals in alliance building and marketing. To that end, effective communication strategies and outreach skills specific to responsible use of social media can provide clear advantages given that these skills and strategies are different and may need modification in those with LHL. It is imperative to ensure the needed knowledge and training for accountable approach to social media is provided to health care providers, so that these outreach strategies are utilized with the needed awareness of their unique strengths and pitfalls, as follows [25]:

- (i) Consumers’ and care partners’ confidentiality may not be breached upon posting anything on social media, including indirect referencing to a specific individual or a particular description of a condition unique to a specific person (e.g., upon soliciting for transplant kidney donors on social media). [26, 27]
- (ii) Confidential information about clinics, hospitals, dialysis centers, or similar health care and advocacy entities may not be disclosed on social media without ensuring that the needed processes, including collecting authorizations to disclose, are undertaken.
- (iii) Health care workers’ job security and careers should remain protected with thorough review of the content of the messages and illustrations/videos before online posting.
- (iv) Careless and disrespectful language and emotional tones are often counterproductive and may not be justified under the context of freedom of speech.

**Table 2:** Social media that are more frequently used for kidney education and advocacy

Social media	Strength	Limitations	Additional comments
Facebook	Frequently used social media platform by many kidney patients and patient groups	Widely used for entertaining purposes, which can dilute its professional utility	User-friendly platform for kidney advocacy, enabling wide ranges of outreach goals
Instagram	Photo-predominating platform	Not frequently used by health care professionals	Picture friendly, potentially effective for illustrative educational purposes
Twitter	Often used by physician specialists and scientists, including nephrologists	Less frequently used by patients and care partners	Increasing popularity among physician and specialty circles
LinkedIn	More often used by professionals, including in industry	Originally designed for employment and job-seeking networking	Mostly effective to reach out to industry and managerial professionals
YouTube	Video-predominating platform	Less effective with non–video-based formats	Wide ranges of outreach and educational targets
WeChat	Widely used in mainland China	Access is often limited to those living in China or its diaspora	Effective platform to reach out to patients and health care professionals in China
Pinterest	Picture-based, often used by dietitians	Currently limited use by some health care workers	Useful for dietary and lifestyle education

Other popular social media at the time of this publication include, but not limited to, Tik Tok, Snapchat, Reddit, Tumblr, Telegram, Quora, and many others that are currently only occasionally used in kidney advocacy activities. Mobile and social media messaging apps include, but not limited to, WhatsApp, Zoom, Facebook Messengers, Skype Teams, and Slack. Note that platforms that are more often used as internet-based messaging are not included

## The global kidney community of policy and advocacy

Policy and advocacy are well-recognized tools that, if properly deployed, can bring about change and paradigm shift at the jurisdictional level. The essence of advocating for policy change to better address kidney disease is, in itself, an exercise in improving health literacy of the policy makers. Policy development, at its core, is a key stakeholder or stakeholder group (e.g., the kidney community, who believes that a problem exists that should be tackled through governmental action). There is an increasing recognition of the importance of formulating succinct, meaningful, and authentic information, akin to improving health literacy, to present to government for action.

Robust and efficacious policy is always underpinned by succinct and applicable information; however, the development and communication of this message, designed to bridge the gap in knowledge of relevant jurisdictions, is only part of the process of policy development. An awareness of the process is important to clinicians who are aiming to advocate for effective change in prevention or improvement of outcomes in the CKD community.

**Fig. 2:**
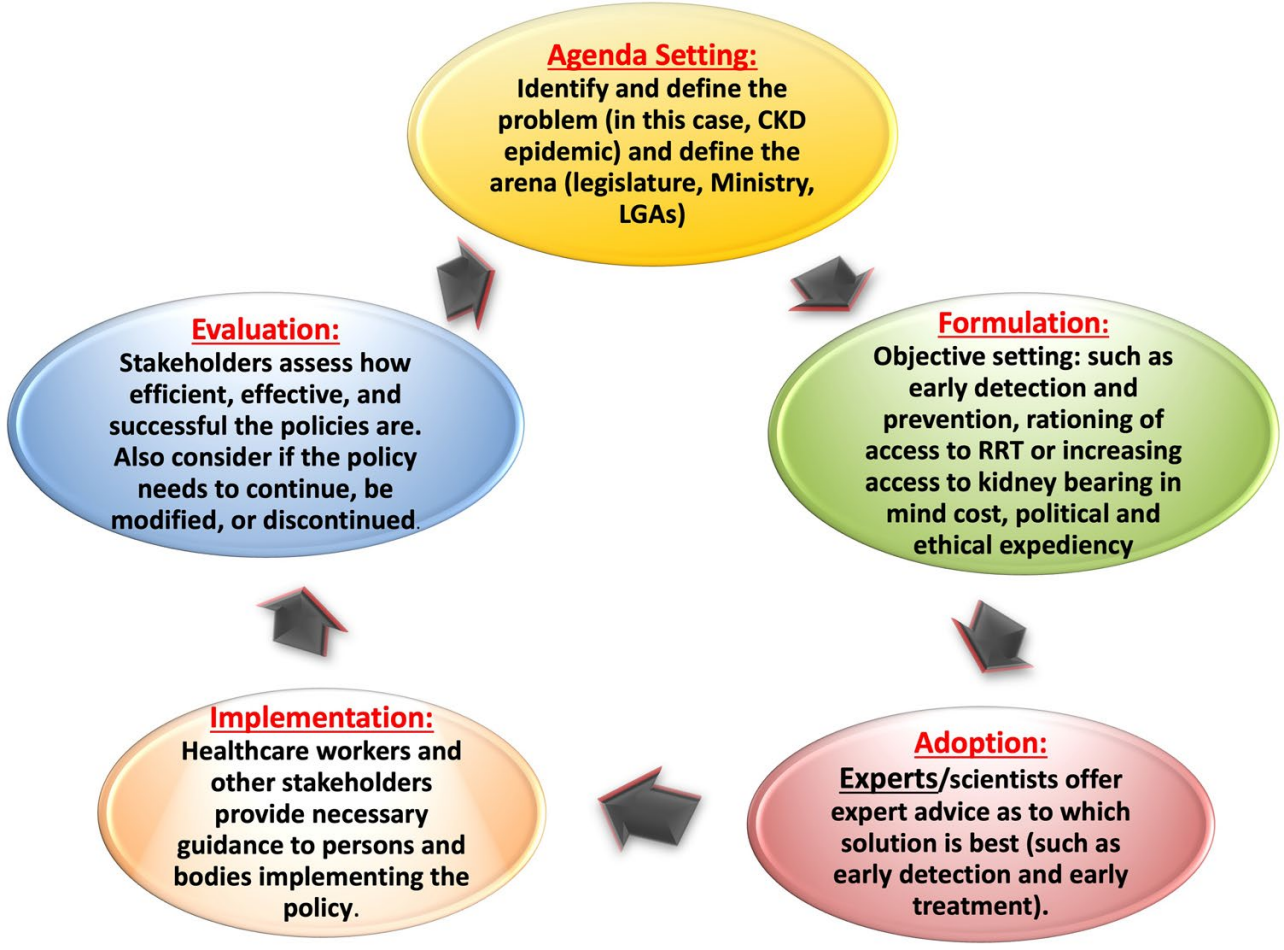
Policy cycle involving five stages of policy development. *CKD* chronic kidney disease, *KRT* kidney replacement therapy, *LGA* local government area

Public policies, the plans for future action accepted by governments, are articulated through a political process in response to stakeholder observation, usually written as a directive, law, regulation, procedure, or circular. Policies are purpose-fit and targeted to defined goals and specific societal problems and are usually a chain of actions effected to solve those societal problems [28]. Policies are an important output of political systems. Policy development can be formal, passing through rigorous lengthy processes before adoption (such as regulations), or it can be less formal and quickly adopted (such as circulars). As already mentioned, the governmental action envisaged by the key stakeholders as solution to a problem is at its core. The process enables stakeholders to air their views and bring their concerns to the fore. Authentic information that is meaningful to the government is critical. The policy development process can be stratified into 5 stages (i.e., the policy cycle), as depicted by Anderson (1994) [29] and adapted and modified by other authors [30] (Fig. 2). The policy cycle constitutes an expedient framework for evaluating the key components of the process.

Subsequently, the policy moves on to the implementation phase. This phase may require subsidiary policy development and adoption of new regulations or budgets (implementation). Policy evaluation is integral to the policy processes and applies evaluation principles and methods to assess the content, implementation, or impact of a policy. Evaluation facilitates understanding and appreciation of the worth and merit of a policy as well as the need for its improvement. More important, of the 5 principles of advocacy that underline policy making [31], the most important for clinicians engaged in this space is that of commitment, persistence, and patience. Advocacy takes time to yield the desired results.

The Advocacy Planning Framework, developed by Young and Quinn in 2002 [30], consists of overlapping circles representing 3 sets of concepts (way into the process, the messenger, and message and activities) that are key to planning any advocacy campaign:

- (i) “Way into the process”: discusses the best approaches to translate ideas into the target policy debate and identify the appropriate audience to target.
- (ii) Messenger: talks about the image maker or face of the campaign and other support paraphernalia that are needed.
- (iii) Message and activities: describe what can be said to the key target audiences that is engaging and convincing. And how best it can be communicated through appropriate communication tools.

Advocacy is defined as "an effort or campaign with a structured and sequenced plan of action which starts, directs, or prevents a specific policy change.” [31] The goal being to influence decision makers through communicating directly with them or getting their commitment through secondary audiences (advisers, the media, or the public) to the end that the decision maker understands, is convinced, takes ownership of the ideas, and finally has the compulsion to act [31]. As with improving health literacy, it is the communication of ideas to policy makers for adoption and implementation as policy that is key. There is much to be done with bridging this gap in understanding of the magnitude of community burden that results from CKD. Without good communication, many good ideas and solutions do not reach communities and countries where they are needed. Again, aligned with the principles of developing resources for health literacy, the approach also needs to be nuanced according to the local need, aiming to have the many good ideas and solutions be communicated to communities and countries where they are needed.

Advocacy requires galvanizing momentum and support for the proposed policy or recommendation. The process is understandably slow as it involves discussions and negotiations for paradigms, attitudes, and positions to shift. In contemplating advocacy activities, multiple factors must be considered, interestingly not too dissimilar to that of building health literacy resources: What obstructions are disrupting the policy-making process from making progress? What resources are available to enable the process to succeed? Is the policy objective achievable considering all variables? Is the identified problem already being considered by the policy makers (government or multinational organizations)? Any interest or momentum generated around it? Understandably, if there is some level of interest and if government already has its spotlight on the issue, it is likely to succeed.

Approaches to choose from include the following [31, 32]:

- Advising (researchers are commissioned to produce new evidence-based proposals to assist the organization in decision making).
- Activism: involves petitions, public demonstrations, posters, fliers, and leaflet dissemination, often used by organizations to promote a certain value set.
- Media campaign: having public pressure on decision makers helps in achieving results.
- Lobbying: entails face-to-face meetings with decision makers; often used by business organizations to achieve their purpose.

Here lies the importance of effective and successful advocacy to stakeholders, including policy makers, health care professionals, communities, and key change makers in society. The WKD, since inception, has aimed at playing this role. WKD has gained people’s trust by delivering relevant and accurate messaging and supporting leaders in local engagement, and it is celebrated by kidney care professionals, celebrities, those with the disease, and their care givers all over the world. To achieve the goal, an implementation framework of success in a sustainable way includes creativity, collaboration, and communication.

The ongoing challenge for the International Society of Nephrology and International Federation of Kidney Foundations–World Kidney Alliance, through the Joint Steering Committee of WKD, is to operationalize how to collate key insights from research and analysis to effectively feed the policy-making process at the local, national, and international levels, to inform or guide decision making (i.e., increasing engagement of governments and organizations, like World Health Organization, United Nations, and regional organizations, especially in low-resource settings). There is a clear need for ongoing renewal of strategies to increase efforts at closing the gap in kidney health literacy, empowering those affected with kidney disease and their families, giving them a voice to be heard, and engaging with the civil society. This year, the Joint Steering Committee of WKD declares *“Kidney Health for All”* as the theme of the 2022 WKD to emphasize and extend collaborative efforts among people with kidney disease, their care partners, health care providers, and all involving stakeholders for elevating education and awareness on kidney health and saving lives with this disease.

## Conclusions

In bridging the gap of knowledge to improve outcomes for those with kidney disease on a global basis, an in-depth understanding of the needs of the community is required. The same can be said for policy development, understanding the processes in place for engagement of governments worldwide, all underpinned by the important principle of codesign of resources and policy that meets the needs of the community for which it is intended.

For World Kidney Day 2022, kidney organizations, including the International Society of Nephrology and International Federation of Kidney Foundations–World Kidney Alliance, have a responsibility to immediately work toward shifting the patient-deficit health literacy narrative to that of being the responsibility of clinicians and health policy makers. LHL occurs in all countries regardless of income status; hence, simple, low-cost strategies are likely to be effective. Communication, universal precautions, and teach-back can be implemented by all members of the kidney health care team. Through this vision, kidney organizations will lead the shift to improved patient-centered care, support for care partners, health outcomes, and the global societal burden of kidney health care.

## Supplementary Information

Below is the link to the electronic supplementary material.

Supplementary file1 (DOCX 134 kb)
