# Supplementary material for: Kidney health for all: bridging the gap in kidney health education and literacy
Source: J Nephrol. 2022 Mar 14;35(6):1555–63. doi: 10.1007/s40620-022-01290-4 (PMC9300568; doi:10.1007/s40620-022-01290-4)
Supplement: Supplementary file 1 — Supplementary file1 (DOCX 134 kb) [file 40620_2022_1290_MOESM1_ESM.docx]

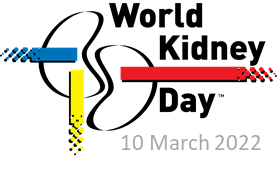

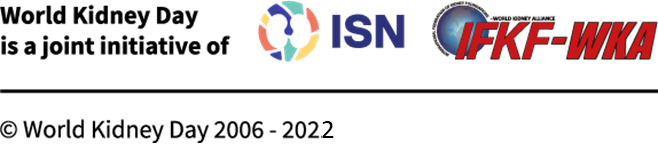


31 January 2022

Re: The World Kidney Day (WKD) 2022 manuscript, “Kidney Health for All”

Dear Editors,

On behalf of the World Kidney Day (WKD) Joint Steering Committee, we are submitting to you directly a manuscript entitled – **‘Kidney Health for All – Bridging the Gap in Kidney Health Education and Literacy’** - the official WKD Editorial for 2022.

The 2022 WKD theme focuses on a patient-centered topic with emphasis on knowledge gaps that pose substantial barriers in concerted efforts to overcome the high burden and complications from kidney disease and promoting global teamwork in advancing strategies in bridging the gap in kidney health education and literacy by motiving all involved parties as well as engaging in and supporting kidney health-centered policy making, community health planning, and patient health literacy,

The editorial is co-written by Drs. Robyn G. Langham, Kamyar Kalantar-Zadeh, Ann Bonner, and Siu-Fai Lui on behalf of the World Kidney Day Steering Committee.

We hope to time the publication of the manuscript with the February or March 2022 issue of your journal, so it will be published close to World Kidney Day on 10^th^ March 2022. Please note that the article is under embargo until 1 February 2022. This article’s first place of publication will be *Kidney International,* and it is being offered to a wide range of medical journals, including leading nephrology, general medicine and other journals for reprint. Therefore, if you accept the article for print in your publication, no substantive changes to the text can be made; only minor stylistic and spelling changes, detail, and length of manuscript in keeping with your journal’s style can be done.

This manuscript is written every year by invited authors working for the campaign on a voluntary basis. They receive neither grants nor prizes yet dedicate their time to the valuable cause that this campaign champions. The World Kidney Day is not in a position to pay any fee; therefore, we are also asking for a fee waiver. As a reflection of the worldwide support for this international event, the WKD 2021 editorial was published in over 40 journals worldwide. We are sure you appreciate why we ask for a complete fee waiver. We hope to count on your journal in 2022.

Please address all correspondence related to this paper to Dr. Ekamol Tantisattamo: [etantisa@hs.uci.edu](mailto:etantisa@hs.uci.edu). We thank you in advance for considering publishing the WKD 2022 manuscript in your journal.

Sincerely,

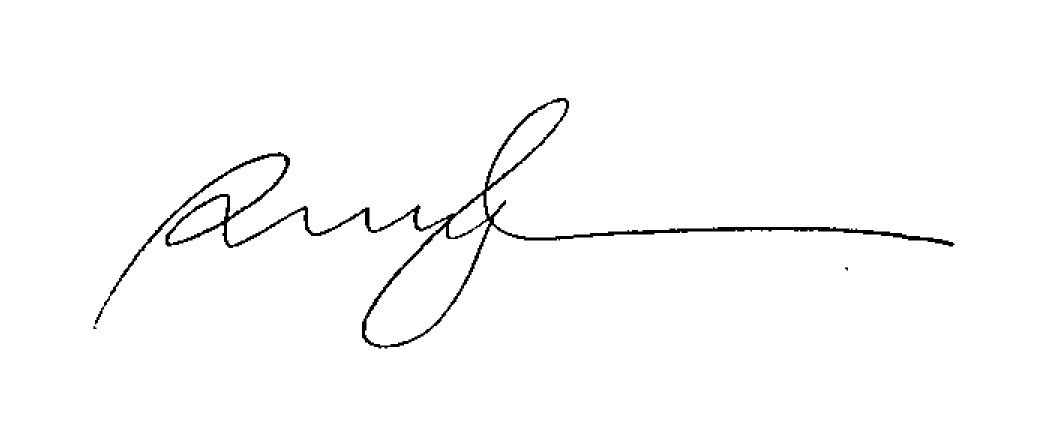


__________________________ _____________________________

Robyn G. Langham, MBBS, PhD Kam Kalantar-Zadeh, MD, MPH, PhD

Prof. Robyn G. Langham Prof. Kamyar Kalantar-Zadeh

International Society of Nephrology International Federation of Kidney Foundations

Co-chair, Joint Steering Committee Joint Steering Committee

World Kidney Day World Kidney Day
